# Supplementary material for: Partial Ablation of Non-Myogenic Progenitor Cells as a Therapeutic Approach to Duchenne Muscular Dystrophy
Source: Biomolecules. 2021 Oct 15;11(10):1519. doi: 10.3390/biom11101519 (PMC8534118; doi:10.3390/biom11101519)
Supplement: Supplementary file 1 [file biomolecules-11-01519-s001.zip › biomolecules-1305363-supplementary.pdf]

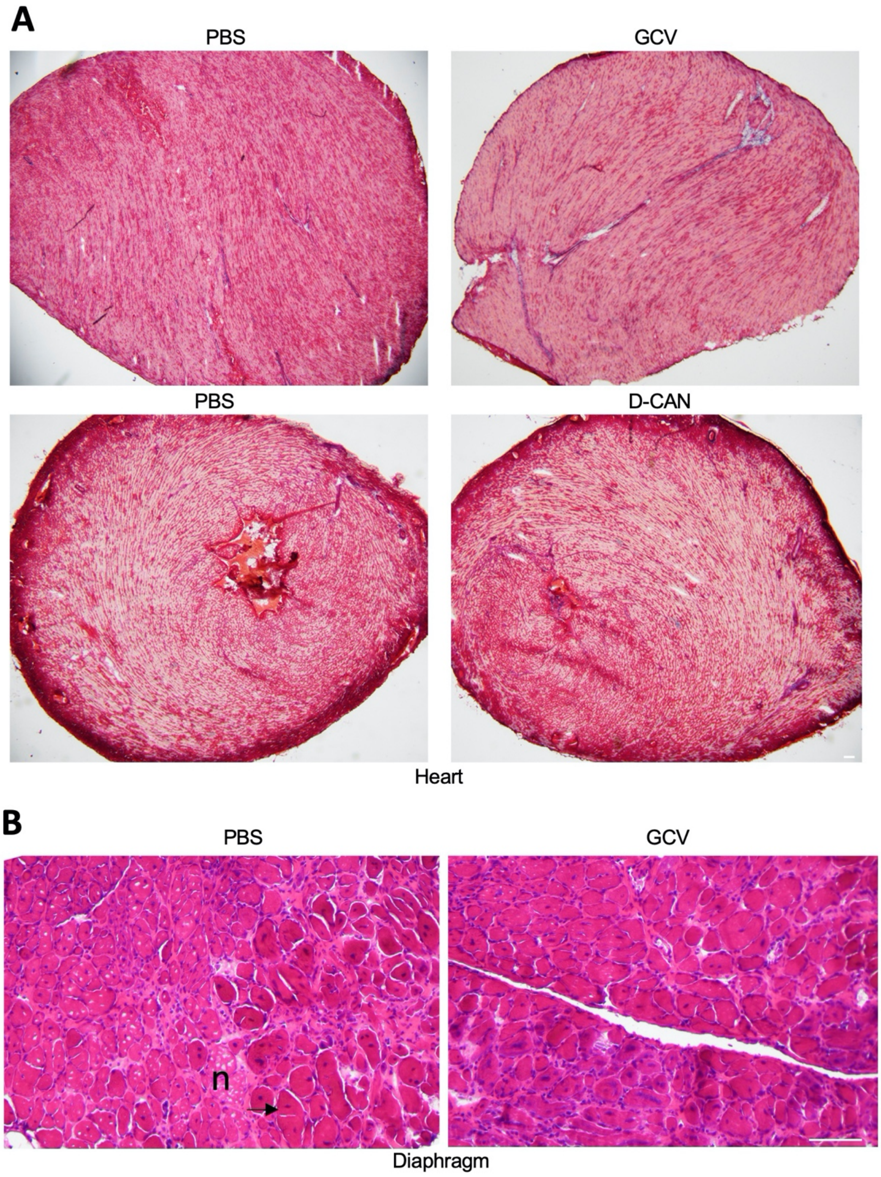

Figure S1. Histological tissue section analysis. (**A**) Low magnification heart Masson's trichrome staining images used for left ventricle high magnification in Figure 3e. and Figure 4c. (**B**) (A) High magnification diaphragm H&E staining from mice analyzed in Figure 3E. n: necrosis. Arrow: myofiber with centrally-localized nuclei. Scale bar: 100 μm.

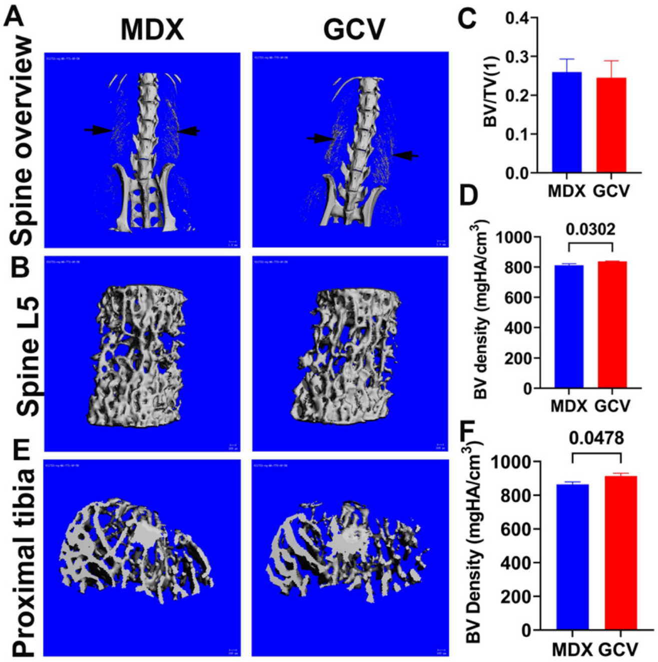

Figure S2. Bone analysis for Figure 3a–c experiment. Mouse lumbar spine and tibia were dissected and fixed in neutral buffered formalin for 48 h. MicroCT scans were performed using a Viva CT 80 (Scanco Medical) with the X-ray energy of 70kVP and 113μA. Micro-CT analysis was performed with a scanning resolution of 15 μm. We analyzed the proximal tibia for trabecular bone and the spine trabecular bone of the entire lumbar 5 (spine L5). We analyzed 50 slices right beneath the growth plate for proximal tibia trabecular bone (metaphysis). We used Gauss = 0.8, Sigma = 1, and threshold = 163 for trabecular bone of the proximal tibia and spine. Trabecular bone analysis was performed by carefully contouring the trabecular part of the proximal tibia and spine L5. All the bone parameters were generated by 3D evaluation software automatically, including bone volume/total volume (BV/TV), bone volume density, trabecular number (Tb.N), trabecular thickness (Tb.Th), and trabecular separation (Tb.Sp).
